# Treatment-related fluctuations in Guillain-Barré syndrome​: clinical features and predictors of recurrence

**DOI:** 10.1590/0004-282X-ANP-2021-0226

**Published:** 2022-02-21

**Authors:** Lucas ALESSANDRO, Juan Ignacio CASTIGLIONE, Patricio BRAND, Veronica BRUNO, Fabio BARROSO

**Affiliations:** 1 Raul Carrea Institute for Neurological Research, Department of Neurology, Buenos Aires, Argentina.; 2 Raul Carrea Institute for Neurological Research, Department of Neuromuscular, Buenos Aires, Argentina.; 3 University of Calgary, Hotchkiss Brain Institute, Department of Clinical Neurosciences, Calgary, Alberta, Canada.

**Keywords:** Cytomegalovirus, Herpesvirus 4, Human, Guillain-Barré Syndrome, Infectious Mononucleosis, Citomegalovirus, Herpesvirus Humano 4, Síndrome de Guillain-Barré, Mononucleosis Infecciosa

## Abstract

**Background:**

A treatment-related fluctuation (TRF) in a patient with Guillain-Barré syndrome (GBS) is defined as clinical deterioration within two months of symptom onset following previous stabilization or improvements with treatment.

**Objective:**

To investigate the clinical characteristics and factors that could increase the risk of relapse of GBS in patients with and without TRFs.

**Methods:**

Retrospective review of medical records of patients (>18 years) with GBS evaluated between January/2006 and July/2019. Demographic and clinical characteristics, ancillary studies, treatment received, and the clinical course of patients with and without TRFs were analyzed.

**Results:**

Overall, 124 cases of GBS were included; seven (5.6%) presented TRFs. GBS-TRF cases were triggered more frequently by infectious mononucleosis (28.57 vs. 8.55%; p=0.01). GBS-TRF were initially treated with plasmapheresis more frequently than those without TRF (14.29 vs. 1.70%; p=0.0349). Combined treatment (71.43 vs. 4.27%; p<0.001) and corticosteroids (42.86 vs. 1.71%; p<0.001) were more commonly used in the GBS-TRF group. GBS-TRF patients presented a higher median initial disability score (4 vs. 2; p=0.01).

**Conclusions:**

Patients with GBS triggered by infectious mononucleosis and a high degree of initial disability have higher chances of developing TRFs. Although patients with TRF were treated with plasmapheresis more often, the total number was too low to suggest a link between plasma exchange and TRF.

## RESUMEN

**Antecedentes:**

Una fluctuación relacionada al tratamiento (FRT) en un paciente con síndrome de Guillain-Barré (SGB) se define como un deterioro clínico dentro de los dos meses posteriores al inicio de los síntomas después de una estabilización previa o mejoría con el tratamiento.

**Objetivo:**

Investigar las características clínicas y los factores que podrían incrementar el riesgo de recaída, comparando pacientes con SGB, con y sin FRT.

**Métodos:**

Revisión retrospectiva de historias clínicas de pacientes (>18 años) con SGB evaluados entre enero/2006 y julio/2019. Se analizaron las características demográficas y clínicas, los estudios complementarios, el tratamiento recibido y la evolución clínica de los pacientes con y sin FRT.

**Resultados:**

Se incluyeron 124 casos de SGB en el total; 7 (5,6%) presentaron FRT. Los casos de SGB con FRT se desencadenaron con mayor frecuencia por mononucleosis infecciosa (28,57 *vs*. 8,55%; p=0,01). Los casos de SGB con FRT se trataron inicialmente con plasmaféresis con más frecuencia que aquellos sin FRT (14,29 *vs*. 1,70%; p=0,0349). El tratamiento combinado (71,43 *vs*. 4,27%; p<0,001) y los corticosteroides (42,86 *vs*. 1,71%; p<0,001) se utilizaron con mayor frecuencia en el grupo de SGB con FRT. Los pacientes con FRT presentaron una escala de discapacidad inicial mediana más alta (4 *vs*. 2; p=0,01).

**Conclusiones:**

Aquellos SGB desencadenados por mononucleosis infecciosa y un alto grado de discapacidad inicial tienen una mayor probabilidad de desarrollar FRT. Aunque los pacientes con FRT fueron tratados con plasmaféresis con mayor frecuencia, el número total fue demasiado bajo para sugerir un vínculo entre la plasmaféresis y FRT.

## INTRODUCTION

**Figure 1.**
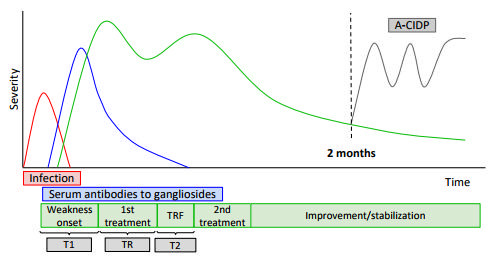
Guillain-Barré definitions and time course: TRF defines the situation when patients with GBS, previously stabilized or improved with treatment, show clinical deterioration within two months of the beginning of the pathology. Those patients who have ≥ 3 TRF or progress clinically after two months from the onset of motor symptoms were excluded and classified as A-CIDP.

A-CIDP: acute-onset chronic inflammatory demyelinating polyneuropathy; GBS: Guillain-Barré syndrome; T1: starting time of first treatment; T2: time from the start of relapse to the beginning of the second treatment; TR: time to relapse; TRF: treatment-related fluctuations.

Treatment-related fluctuation (TRF) in patients with Guillain-Barré syndrome (GBS) describes those cases in which clinical deterioration occurs one or more times after initial improvement or stabilization with intravenous immunoglobulin (IVIg) or plasmapheresis (PE) treatment within the first two months after the onset of symptoms (Figure 1). This phenomenon affects 5-26% of patients with GBS^1^ ^,^ ^2^ ^,^ ^3^ ^,^ ^4^ ^,^ ^5^ ^,^ ^6^ ^,^ ^7^. Currently, the underlying physiological processes and risk factors related to TRFs are unknown. These mechanisms could vary according to geographical area^8^, due to the different clinical phenotypes or electrophysiological subtypes.

The objective of our work was to investigate the clinical characteristics and factors that could increase the risk of relapse by comparing patients with GBS with and without TRFs.

## METHODS

Clinical charts of consecutive patients aged ≥18 years and diagnosed with GBS between January 2006 and July 2019 in our center (FLENI, Buenos Aires, Argentina) were retrospectively analyzed. The study was approved by the institutional ethics committee.

Demographic characteristics and past medical history were recorded. Infectious symptoms or vaccine administration occurring up to one-month before symptom onset were considered disease triggers. Infectious mononucleosis was considered in cases of fever, pharyngitis, and adenomegaly due to Epstein-Barr virus (EBV), cytomegalovirus (CMV), or HIV.

Clinical manifestations assessed were pain, motor and sensory deficits, autonomic dysfunction, and cranial nerve involvement. The Medical Research Council-Sum Score (MRC-Sum Score)^3^ was used to assess motor alterations. Autonomic dysfunction was only considered when clinical precipitants that could explain these manifestations were ruled out. An initial cardiovascular evaluation was performed in all patients until clinical stability was verified. Hemodynamic monitoring was performed four times a day in clinically stable patients, while those unstable remained in the intensive care unit (ICU) under continuous cardiovascular monitoring.

Lumbar puncture (LP) and cerebrospinal fluid (CSF) analysis were performed in all patients during acute illness. Albuminocytologic dissociation was considered present when CSF-protein level was increased (>45 mg/dL), without CSF-pleocytosis (leukocyte count of <10/mm^3^).

All patients underwent electrophysiological nerve conduction studies at least once within the first four weeks from the onset of symptoms. Amplitude and conduction velocity were measured after stimulation at conventional sites^9^. Electrophysiological patterns were classified as demyelinating, axonal, or undetermined/normal^10^.

Established treatment and delay in administration were analyzed. Disability was assessed at admission, and 6 and 12 months using the GBS Disability Scale proposed by Hughes^11^.

A TRF was defined as^5^: a) improvement in the GBS disability score of at least one grade or improvement in the MRC sum score of more than 5 points after treatment completion, followed by a decline in the GBS disability score of at least one grade or a reduction in the MRC sum score of more than 5 points within the first month after the onset of symptoms; or b) steady clinical course for more than one week after treatment completion, followed by a decline of at least one grade of the GBS disability score or more than 5 points on the MRC sum score.

Patients who had ≥3 TRFs or progressed clinically after two months from the onset of motor symptoms were excluded and classified as Acute-onset Chronic Inflammatory Demyelinating Polyneuropathy (A-CIDP)^5^.

Differences in demographic and clinical characteristics, ancillary studies, treatment, and clinical course between both groups were compared using the Wilcoxon rank-sum test (Stata13v). An alpha value of 0.05 was considered statistically significant.

## RESULTS

**Figure 2.**
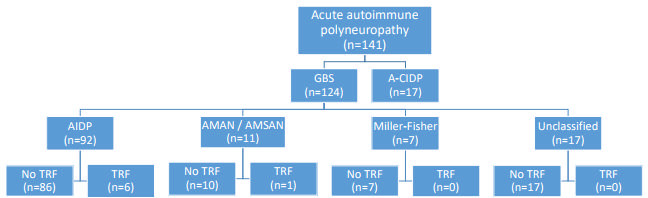
Flowchart of patient inclusion.

A-CIDP: acute-onset chronic inflammatory demyelinating polyneuropathy; AIDP: acute inflammatory demyelinating polyneuropathy; AMAN: acute motor axonal neuropathy; AMSAN: acute motor-sensory axonal neuropathy; GBS: Guillain-Barré Syndrome; TRF: treatment-related fluctuations.

A total of 124 patients with GBS were included. Seven patients (5.64%) presented TRFs, with a median age of 53 years (range 32-81 years) and a slight male predominance (57.14%). There were no significant differences in sex and age among GBS patients with and without TRFs (Table 1). A-CIDP patients were excluded (Figure 2). Patients with subacute inflammatory demyelinating polyneuropathy (SIDP) and other variants of CIDP were also excluded.

**Table 1.** Comparison between GBS patients with and without treatment-related fluctuations.

	Total (n=124)	GBS without TRF (n=117)	GBS with TRF (n=7)	p-value
Demographic data				
Age in years; median (range)	48 (21-86)	48 (21-86)	53 (32-81)	0.51
Men; n (%)	71 (57.26)	67 (57.26)	4 (57.14)	0.99
Diabetes mellitus; n (%)	11 (8.87)	9 (7.69)	2 (28.57)	0.06
Autoimmune disease; n (%)	28 (22.58)	27(23.08)	1 (14.29)	0.59
Facial paralysis; n (%)	8 (6.45)	7 (5.98)	1 (14.29)	0.38
Oncological disease; n (%)	10 (8.06)	9 (7.69)	1 (14.29)	0.53
HIV; n (%)	5 (4.07)	5 (4.31)	0	0.57
*Triggers; n (%)*	86 (69.35)	82 (70.08)	4 (57.14)	0.47
Respiratory infection; n (%)	41(33.06)	40 (34.19)	1 (14.29)	0.27
Acute diarrhea; n (%)	31 (25.00)	30 (25.64)	1 (14.29)	0.50
Infectious mononucleosis; n (%)	8 (6.45)	6 (5.12)	2 (28.57)	0.01*
Vaccination; n (%)	2 (1.61)	2 (1.71)	0	0.72
Others; n (%)	4 (3.22)	4 (3.41)	0	0.62
Time from trigger to the first symptom; median days (range)	10 (1-30)	10 (1-30)	5 (3-10)	0.10
Time from trigger to weakness; median days (range)	11(1-40)	11(1-40)	7(3-37)	0.48
Clinical manifestations				
Neuropathic pain; n (%)	78 (62.9)	71 (60.68)	7 (100)	0.03*
Neck MRC; median (range)	5 (0-5)	5	4	0.86
Worst MRC-Sum Score; median(range)	51 (0-60)	51	50	0.78
*Sensory disturbances; n (%)*	110 (88.71)	104 (88.89)	6 (85.71)	0.79
Paresthesia; n (%)	94 (77.69)	87 (76.32)	7 (100)	0.14
Cutaneous sensory; n (%)	50 (41.32)	46 (40.35)	4 (57.14)	0.38
Proprioceptive sensory; n (%)	44 (36.36)	42 (36.84)	2 (28.57)	0.66
Sensory ataxia; n (%)	25 (20.16)	23 (19.66)	2 (28.57)	0.56
*Osteotendinous reflexes*				
Generalized areflexia; n (%)	66 (53.23)	61 (52.14)	5 (71.43)	0.32
Hyporeflexia/partial areflexia; n (%)	50 (40.32)	48 (41.03)	2 (28.57)	0.51
Normal; n (%)	8(6.45)61	8(6.48)	0	0.47
Autonomic dysfunctions; n (%)	43 (34.68)	40 (34.19)	3 (42.86)	0.64
Severe; n (%)	21 (16.94)	20 (17.09)	1 (14.29)	0.84
HR abnormality; n (%)	30 (24.19)	28 (23.93)	2 (28.57)	0.78
BP changes; n (%)	26 (20.97)	23 (19.66)	3 (42.86)	0.14
Gastrointestinal disturbances; n (%)	18 (14.52)	15 (12.82)	3 (42.86)	0.02*
Genitourinary disturbances; n (%)	15 (12.10)	14 (11.97)	1 (14.29)	0.85
Temperature dysregulation; n (%)	10 (8.06)	8 (6.84)	2 (28.57)	0.04*
*CN involvement; n (%)*	60 (48.39)	57 (48.72)	3 (42.86)	0.76
Ocular-motility; n (%)	11 (8.87)	10 (8.55)	1 (14.29)	0.60
Facial; n (%)	54 (43.55)	52 (44.44)	2 (28.57)	0.41
Bilateral facial; n (%)	30 (24.19)	28 (23.93)	2 (28.57)	0.78
Lower CN; n (%)	10 (8.06)	9 (7.69)	1 (14.29)	0.53
Multiple CN involvement; n (%)	15 (12.12)	14 (11.97)	1 (14.29)	0.85
CSF and electrophysiological variant characteristics				
Albuminocytologic dissociation; n (%)	85 (69.67)	79 (68.70)	6 (85.71)	0.34
CSF protein level; mg/dL median (range)	79.9 (38.6-605)	79.9 (38.6-605)	90.94 (60-176)	0.47
Time between symptom onset and LP in days; median (range)	7 (1-45)	7 (1-45)	7(2-30)	0.60
*EMG classification*				
AMAN; n (%)	4 (3.23)	4 (3.23)	0	0.62
AMSAN; n (%)	7 (5.65)	6 (5.13)	1 (14.29)	0.30
AIDP; n (%)	89 (71.77)	83 (70.94)	6 (85.71)	0.40
Unclassified/Normal; n (%)	18 (14.52)	18 (15.38)	0	0.26
Miller Fisher; n (%)	6 (4.84)	6 (5.13)	0	0.54
Treatment and evolution				
No treatment requirements; n (%)	9 (7.26)	9 (7.69)	0	0.44
Treatment establishment time in days; median in days (range)	7 (1-45)	7 (1-45)	7(2-30)	0.64
*Initial treatment*				
Immunoglobulin; n (%)	112 (90.32)	106 (90.59)	6 (85.71)	0.67
Plasmapheresis; n (%)	3 (2.41)	2 (1.70)	1 (14.29)	0.0349*
Combined treatment; n (%)	10 (8.06)	5 (4.27)	5 (71.43)	<0.001*
Corticosteroids; n (%)	5 (4.03)	2 (1.71)	3 (42.86)	<0.001*
Admission at ICU; n (%)	18 (14.52)	16 (13.68)	2 (28.57)	0.27
Days in ICU; median (range)	0 (0-120)	0 (0-120)	0 (0-10)	0.38
Orotracheal intubation; n (%)	10 (8.06)	9 (7.69)	1 (14.29)	0.53
Non-invasive ventilation; n (%)	5 (4.07)	5 (4.31)	0	0.57
Hemodynamic support; n (%)	6 (4.84)	5 (4.27)	1 (14.29)	0.23
Baseline GBS disability score; median (range)	2 (1-5)	2	4	0.01*
GBS disability score after 6 months; median (range)	0 (0-4)	0	1	0.08
GBS disability score after 1 year; median (range)	0 (0-4)	0	0	0.86
MRC-Sum Score at discharge; median (range)	56 (19-60)	56	53	0.84
Final MRC-Sum Score; median (range)	60 (20-60)	60	60	0.70
Mortality; n (%)	1 (0.81)	1 (0.85)	0	0.80
Follow-up in months; median (range)	36 (6-156)	36 (6-132)	27 (6-156)	0.47

AIDP: acute inflammatory demyelinating polyneuropathy; AMAN: acute motor axonal neuropathy; AMSAN: acute motor-sensory axonal neuropathy; BP: blood pressure; CSF: cerebrospinal-fluid; CN: cranial nerve; GBS: Guillain-Barré Syndrome; HR: heart rate; ICU: intensive care unit; LP: Lumbar puncture; MRC: Medical Research Council; TRF: treatment-related fluctuations.

There were no significant differences in past medical history or percentage of patients who had infectious triggers before GBS. However, those with TRFs had a higher frequency of infectious mononucleosis before GBS (28.57 vs. 8.55%; p=0.01) (Table 1). One of the cases was secondary to cytomegalovirus (CMV), while the other was preceded by an Epstein Barr Virus (EBV) infection.

Neuropathic pain was the most frequent clinical manifestation in patients with TRFs (100 *vs.* 60.68%; p=0.03). There were no significant differences in muscle power, sensory deficits, and cranial nerve involvement between the two groups. Dysautonomia was slightly more frequent in patients with TRFs, although the difference was not significant (85.71 *vs.* 68.70%; p=0.34) (Table 1). However, patients with TRFs had a higher frequency of gastrointestinal disorders (42.86 *vs.* 12.82%; p=0.02) and temperature dysregulation (28.57 *vs.* 6.84%; p=0.04).

There were no differences in CSF findings between GBS patients with and without TRFs. The most frequent electrophysiological presentation in patients with TRFs was acute inflammatory demyelinating polyneuropathy -AIDP- (85.71%); only one patient presented an axonal variant. There were no electrophysiological differences between the two groups (Table 1).

Six (85.71%) patients with TRFs received IVIg as the first treatment at a dose of 2 g/kg administered on five consecutive days. Only one (14.29%) received PE (seven sessions) (Table 2). The median delay in treatment administration from symptom onset was seven days (range 3-30 days), without differences between the two groups. The median time to first relapse was nine days (range 2-15 days). Only two patients (#4 and #5) switched the initial treatment. In three cases (#1, #6, and #7), corticosteroids were added to the IVIg scheme. The median delay in starting the second treatment was 16 days (2-25 days). Only one patient had more than one relapse. The median total recovery time from symptom onset was 44 days (range 30-60 days). Patients with TRFs received PE more frequently than those without TRFs (14.29 *vs.* 1.70%; p=0.0349). Furthermore, patients with TRFs more frequently required a combination of treatments (71.43 *vs.* 4.27%; p<0.001) and the addition of corticosteroids (42.86 *vs.* 1.71%; p<0.001).

**Table 2.** Initial and relapse treatment.

N°/Age/Sex	T_1_ (days)	Initial treatment	MRC_1_/D_1_	T_R_ (days)	T_2_ (days)	Relapse treatment	Total n° of TRF	TT (days)	MRC_2_/D_2_	MRC_3_/D_3_
1/33/M	4	IVIg (2 g/kg)	45/4	4	16	1st relapse: IVIg (2 g/kg) 2nd relapse: corticosteroids	2	60	60/1	60/0
2/32/M	9	IVIg (2 g/kg)	52/2	14	2	IVIg (2 g/kg)	1	35	60/0	60/0
3/71/F	7	IVIg (2 g/kg)	58/3	30	20	IVIg (1 g/kg)	1	60	59/2	60/1
4/81/F	14	IVIg (2 g/kg)	52/4	2	25	PE (5 sessions)	1	44	53/ 2	-
5/53/M	2	PE (7 sessions)	39/5	15	23	IVIg (2 g/kg)	1	38	54/ 2	60/0
6/57/F	30	IVIg (2 g/kg)	50/3	4	8	IVIg (2 g/kg)+corticosteroids	1	54	60/1	60/1
7/46/M	3	IVIg (2 g/kg)	50/4	9	8	IVIg (2 g/kg)+corticosteroids (4 months)	1	30	58/1	60/0

D_1_: GBS disability scale prior to treatment; D_2_: GBS disability scale at 6 months; D_3_: GBS disability scale at 12 month; F: female; IVIG: intravenous immunoglobulin; M: male; MRC_1_: Medical Research Council prior to treatment; MRC_2_: Medical Research Council at 6 months; MRC_3_: Medical Research Council at 12 months; PE: plasmapheresis; T_1_: start time of first treatment; T_2_: time from the start of relapse to the start of the second treatment; T_R_: time to relapse; T_T_: total time to stabilization or improvement; TRF: treatment-related fluctuations.

Patients with TRFs had higher baseline disability scores (4 *vs.* 2; p=0.01), with a slight non-significant difference at six months (1 *vs.* 0; p=0.08) (Table 1). There were no significant differences in the MRC-Sum Score (initial and final), ICU admissions, and mortality between groups.

## DISCUSSION

The frequency of TRFs in patients with GBS varies according to different series^1^ ^,^ ^2^ ^,^ ^3^ ^,^ ^4^ ^,^ ^5^ ^,^ ^6^ ^,^ ^7^. Original reports with fewer patients suggested that TRFs could occur in up to 26% of GBS cases^1^. Yet, some of those reports^2^ ^,^ ^3^ included patients who would be currently characterized as A-CIDP^5^. The most recent series published by the International GBS Outcome Study (IGOS), which is multicenter and has a substantial sample size^7^, showed that the prevalence of TRFs is low (5%). In our series, we observed that seven (5.64%) of 124 adult patients presented TRFs, in agreement with the results from the IGOS.

Demographic characteristics and past clinical history were similar between the GBS groups with and without TRFs (Table 1). Interestingly, the GBS group with TRFs was more frequently preceded by infectious mononucleosis secondary to CMV and EBV (28.57 *vs.* 8.55%; p=0.01) and showed higher baseline disability scores (4 *vs.* 2; p=0.01). It has been demonstrated that patients with GBS triggered by CMV and EBV show more severe clinical manifestations and a greater degree of disability^12^. This could be related to the fact that the immune response mounted after the infection of these viruses causes a higher concentration of molecules associated with the activation and migration of “T” cells^12^. However, in that report, no increased risk of TRF was observed when GBS was preceded by infection with these viruses^12^.

On the other hand, other infectious triggers and electrophysiological variants of GBS are less associated with TRFs. GBS triggered by diarrhea, which causes axonal variants with anti-GM1 antibodies, had a lower frequency of TRFs^4^. Our population showed similar observations since only one patient with RFT had diarrhea as an infectious trigger and one patient had an axonal variant (Table 1).

The initial clinical manifestations of GBS patients with and without TRF were similar. Nevertheless, neuropathic pain was reported by all cases of TRF. Most previous reports on TRF did not consider pain as a manifestation^1^ ^,^ ^2^ ^,^ ^3^ ^,^ ^4^ ^,^ ^5^ ^,^ ^6^. Only the work by Ruts et al. showed that pain is frequent in patients with TRF (81%)^7^. Although there were no significant differences in symptoms of dysautonomia between the two populations, patients with TRFs more often showed gastrointestinal manifestations and temperature dysregulation. None of the previously mentioned literature reports have evaluated dysautonomic complications in patients with TRFs^1^ ^,^ ^2^ ^,^ ^3^ ^,^ ^4^ ^,^ ^5^ ^,^ ^6^ ^,^ ^7^.

Immunotherapy with IVIg and PE are currently the most effective treatments for GBS^13^ ^,^ ^14^ ^,^ ^15^ ^,^ ^16^ ^,^ ^17^. Both treatments have similar efficacy in reducing disability^15^ ^,^ ^16^. In our series, patients with TRF were more commonly treated with PE than non-TRF patients (14.29 *vs.* 1.70%; p=0.0349). The original study on TRFs was in GBS patients treated only with PE^1^ ^,^ ^2^. These reports attributed TRFs to the fact that immunogenic factors may decrease early during PE but rise again after the completion of such treatment. However, subsequent work showed no significant differences in the frequency of TRFs between patients treated with PE *vs.* IVIg^3^ ^,^ ^4^. Hence, given the small number of GBS patients treated with PE in our series (2.4%), we cannot confirm or reject the hypothesis that TRFs more frequently occur in patients treated with PE.

There is insufficient evidence for the re-treatment of patients with GBS and TRFs^7^ ^,^ ^17^. Still, physicians often choose to re-treat severe fluctuations^7^ ^,^ ^17^. In our series, all patients with TRF were re-treated (Table 2). The treatment start time was similar in both groups (median of seven days), so early treatment was not decisive in relapse. In two cases, the initial treatment was changed; IVIg treatment was repeated in five patients (Table 2). There is insufficient evidence that the combination of IVIg and PE is effective^7^ ^,^ ^8^ ^,^ ^15^ ^,^ ^17^ and that a second IVIg course can be used, although there are prospective works in progress^18^. Besides, three patients (42.86%) with TRFs received empirical corticosteroids in addition to IVIg treatment. It should be noted that the reason why some patients received corticosteroids temporarily was that we initially doubted whether they were not patients with A-CIDP. It is known that corticosteroid treatment is considered ineffective for GBS patients^19^. However, some studies show slight benefits with the addition of corticosteroids to IVIG. Nevertheless, due to the retrospective characteristics of our work, it is not possible to attribute a beneficial effect to treatment with corticosteroids.

In conclusion, in our series, patients with GBS preceded by infectious mononucleosis and who presented a higher degree of initial disability were at a higher risk of developing TRFs. Patients with TRFs were treated with PE more frequently than those without TRFs. Given the small number of patients treated with PE, we cannot affirm that the risk of TRFs increases with its use.
